# Job Demands and Resources as Predictors of Burnout Dimensions in Special Education Teachers

**DOI:** 10.3390/ejihpe15120258

**Published:** 2025-12-15

**Authors:** Vesna R. Jovanović, Čedo Miljević, Darko Hinić, Dragica Mitrović, Slađana Vranješ, Biljana Jakovljević, Sanja Stanisavljević, Ljiljana Jovčić, Katarina Pavlović Jugović, Neda Simić, Goran Mihajlović

**Affiliations:** 1 Academy for Applied Studies Belgrade, Higher Education School of Professional Health Studies, 11000 Belgrade, Serbia; vesna.jovanovic@assb.edu.rs (V.R.J.); biljana.jakovljevic@assb.edu.rs (B.J.); sanja.stanisavljevic@assb.edu.rs (S.S.); ljiljana.jovcic@assb.edu.rs (L.J.); katarina.pavlovic.jugovic@assb.edu.rs (K.P.J.); 2 Institute of Mental Health, Faculty of Medicine, University of Belgrade, 11000 Belgrade, Serbia; cedo.miljevic@imh.org.rs; 3 Faculty of Science, Faculty of Philology and Arts, University of Kragujevac, 34000 Kragujevac, Serbia; 4 Department of Physical Medicine and Rehabilitation, Zvezdara University Hospital, 11000 Belgrade, Serbia; mitdragica@gmail.com; 5 University Clinical Centre of Republic of Srpska, 78000 Banja Luka, Bosnia and Herzegovina; sladjana.vranjes@kc-bl.com; 6 Faculty of Medical Sciences, University of Kragujevac, 34000 Kragujevac, Serbia; neda.simic@fmn.kg.ac.rs (N.S.); goran.sm@eunet.rs (G.M.)

**Keywords:** burnout dimensions, special educational needs, teachers, job demands/resources

## Abstract

Background/Objectives. ICD–11 classifies burnout as a work-related issue arising from chronic workplace stress that has not been successfully managed. According to the Job Demands/Resources Model, job demands represent sources of stress and job resources may buffer the impact of job demands on job strain. Since every profession has its specific spectre of work demands/resources related to stress development, the aim of this study was to examine a model predicting workplace burnout dimensions (emotional exhaustion—EE, depersonalisation—DP, personal accomplishment—PA) in special educational needs (SEN) and general education (GE) teachers, with job demands representing potential “risk factors” and job resources potential “protective factors”. Methods. The study involved 116 SEN teachers from eight primary schools for children with learning difficulties and a sample of 145 teachers from general primary schools in the Belgrade region, which was balanced according to the representation of the main demographic variables in the SEN group. The Maslach Burnout Inventory and Job Characteristics Questionnaire were the instruments employed. Results. No difference was found between SEN and GE teachers in the intensity of burnout dimensions. In the SEN group, Changes were the predictors of all three burnout dimensions, Work environment for EE and DP, Emotional demands and Support from colleagues for EE, Cognitive/Quantitative for PA, and Job control for PA. Concerning the GE group, Support from colleagues predicted all three dimensions, Job control EE and DP, Cognitive/Quantitative DP and PA, Changes DP, and Role conflict and Seniority EE. Conclusions. The results of the study provide a foundation for further testing of a hypothetical predictive model of burnout with job demands as direct predictor and job resources as mediators of this relation.

## 1. Introduction

Burnout syndrome is a phenomenon that is acquiring more and more significance in research and workplace environments. It has been classified in the ICD–11 as an occupational phenomenon and work-related problem arising from “chronic workplace stress that has not been successfully managed” (World Health Organization, 2025). According to the said classification and one of the earliest operationalisations of the concept (Maslach & Leiter, 2022), burnout is characterised by three dimensions: feelings of physical and emotional energy exhaustion (Emotional exhaustion), feelings of negativism related to one’s job and a sense of detachment from people in the workplace (cynicism or Depersonalisation^1^), and a sense of ineffectiveness and reduced competence (reduced Personal accomplishment). Burnout occurs when an employee feels emotional exhaustion under the influence of disadvantageous conditions of their work environment, which in turn makes them perceive their activities as useless for the persons they were meant to help (Maslach & Leiter, 1997, 2022; Maslach et al., 2001). Finally, this may lead to a reduced sense of personal accomplishment, along with blaming clients or themselves for failures at work (Maslach et al., 2001; Maslach & Leiter, 2022). Although workplace burnout is an organisational issue, it affects employees’ overall health, their relationship with their family and friends, and their approach to work; it reduces workplace productivity and has negative effects on the company or society (V. R. Jovanović et al., 2021; Maslach & Leiter, 2022).

Development of burnout syndrome is reliant on the interplay between various factors, from situational/organisational (e.g., working conditions, work overload, job autonomy), social (e.g., social support), to individual (personality traits, stress-coping strategies) (V. R. Jovanović et al., 2021; Maslach & Leiter, 1997; Maslach et al., 2001). For example, there is evidence indicating that certain professions, especially those involving direct contact with people, such as health-care workers, social workers, psychologists, teachers, etc., are more likely to be at risk of burnout (V. R. Jovanović et al., 2021; Maslach et al., 2001; Maslach & Leiter, 2022). Studies reporting a growing presence of stress in the teaching profession (Brunsting et al., 2014; V. Jovanović et al., 2019; Kourmousi & Alexopoulos, 2016) have made researchers direct their attention to investigating specificities of professions and work environments that can act as stressors but also as protective factors for negative outcomes in the workplace.

### 1.1. Job Demands and Job Resources in the Teaching Profession

According to the Job Demands/Resources Model (JD–R), *job demands* represent sources of stress that are connected with different physiological or psychological costs (Bakker & Demerouti, 2007; Demerouti & Bakker, 2011), the most frequent ones in the teaching profession being time pressure, learners’ misbehaviour, conflicts with colleagues, lack of administrative support, and unclear roles (Hlado & Harvankova, 2024; Hlado et al., 2025; Skaalvik & Skaalvik, 2018). *Job resources* constitute positive aspects of work that diminish the effects of work-related stressors (Demerouti & Bakker, 2011; Bakker et al., 2005), the most commonly investigated ones being teacher autonomy, supportive relationship with their colleagues, school management and school governing board, relationship with learners’ parents, and opportunities to acquire knowledge and advance in their career (Skaalvik & Skaalvik, 2018).

The JD–R model holds that welfare of an employee stems from *a balance* between available resources and job demands (Bakker et al., 2005), and that employees facing evolving job demands experience more severe symptoms of declining work efficiency, whereas those having more job resources at their disposal are more motivated to work (Baka, 2015; Radošević et al., 2018). Job resources have motivational potential and encourage higher engagement and positive work-related outcomes (e.g., job performance, creativity, loyalty to the company, service quality and intention to stay) (Hlado et al., 2025). Nonetheless, job demands that go beyond individual abilities and/or are accompanied by lower levels of job resources can cause mental and physical exhaustion. Consequently, this may result in reduced work capacity and also the development of health issues.

### 1.2. Risk Factors

The most frequent sources of stress in the teaching profession come from teacher’s facing learning difficulties and misbehaviour, poor relationship with their colleagues, lack of administrative support, tight deadlines, and unfulfilled expectations (Hlado et al., 2025; V. Jovanović et al., 2019; Skaalvik & Skaalvik, 2018). Moreover, research suggests a significant correlation between cooperation with parents and burnout syndrome, primarily in terms of emotional exhaustion and a sense of personal accomplishment (Drobac & Micic, 2025; Mitchell, 2014; Squillaci & Hofmann, 2021).

Some job-related stressors, such as job dynamics, can have a stimulating function (Baka, 2015), while others, such as role conflict and organisation restrictions, are almost always accompanied by negative feelings that affect employee’s well-being. *Workload* may be manifested through various aspects of work, like emotional, cognitive, quantitative, and physical demands, as well as through requirements of the school head, colleagues, students and parents (Nagy & Takács, 2017). Tight deadlines and unbalanced job requirements have been the subject matter of numerous studies investigating workplace burnout (Maslach et al., 2001; Nagy & Takács, 2017). Certain authors maintain that teachers’ workload contributes greatly to their dissatisfaction, even when the teacher is overall satisfied with their job (Ketheeswarani, 2018).

There are data underlining different aspects of work that impact teachers based on where they work—in general schools or special educational needs (SEN) schools. SEN teachers work with learners with developmental disabilities, which requires constant and intensive contact, as well as an individualised approach. In addition, they frequently find themselves in situations where they must cater to students’ primary biological needs. However, feedback on accomplished learning outcomes is often missing, which can make teachers feel less successful and reduce their sense of personal accomplishment (V. Jovanović et al., 2019; Küçüksüleymanoğlu, 2011). That can be one of the reasons why burnout syndrome is more frequently found in SEN teachers (V. Jovanović et al., 2019). Nevertheless, this feeling may occur with teachers in general schools as well, given that educational reforms are directed at inclusion of a growing number of children with developmental disabilities in general schools worldwide as well as in Serbia (Ministry of Education, 2022). It comes as no surprise, therefore, that certain studies show SEN teachers and general education (GE) teachers to be exposed to similar stressors in the workplace (Plichta, 2014).

Research in Irish secondary schools indicate that work environment also has a prominent role in predicting teachers’ emotional exhaustion, even when controlled for the influence of individual variables (Foley & Murphy, 2015). A five-year observational study with Finnish SEN teachers showed that after five years, perceived work environment fit was still a predictor of exhaustion in these teachers, their cynicism towards colleagues, along with inadequate student–teacher relations (Soini et al., 2019). Concerning work roles, *role conflict* occurs when job requirements become incompatible, and role ambiguity occurs when adequate data, which would enable successful completion of the job, are missing or are not explained (Maslach et al., 2001; Garwood et al., 2018). SEN teachers are exposed to stressors since they perform various roles during their work: teaching their students social skills and the subject matter content, assessing students, developing an individual education plan (IEP), providing expert support to GE teachers teaching students with developmental difficulties, and counselling parents (Garwood et al., 2018). *Changes* also represent a burnout risk factor associated with job roles in a practical sense because role complexity of the teaching job brings about changes at work, and contrariwise. Even teachers satisfied with their job have their limitations in terms of how many changes they can cope with (Salmela-Aro et al., 2019).

### 1.3. Protective Factors

The most prominent protective factors/resources for burnout are social support and work autonomy (Bakker & Demerouti, 2007; Radošević et al., 2018; Salmela-Aro et al., 2019). *Social support* in the workplace represents a set of activities with colleagues and managers that provide certain aspects of assistance (emotional support, mentorship, etc.) (De Stasio et al., 2017; Smetackova et al., 2019; Skaalvik & Skaalvik, 2016). A system of support can function as a “security network”, which makes employees feel less threatened by stressful situations in the workplace, whereby the availability of social support is often more important than the support itself (Plichta, 2014). *Job autonomy/control* is also a significant job resource which relates to the degree of freedom to make decisions, such as timetables and ways of doing work. Concerning the teaching profession, job control is considered from the classroom management perspective of carrying out teaching activities, where teachers have the freedom to select the teaching approach in class; however, the education system is governed by numerous predetermined governmental and ministerial rules that limit teachers’ freedom to make decisions (Plichta, 2014). Control imbalance occurs when employees do not have sufficient control over available resources necessary for their work or authorisation to carry on with their work in the way they find the most effective. Imbalance in job control is most often associated with burnout at the level of personal accomplishment in the workplace (Maslach & Leiter, 1997). Research conducted with Portuguese teachers showed support from colleagues and autonomy to be the key mediating variables between job demands and teachers’ well-being, which suggests that job resources can protect teachers from negative consequences of job demands, alleviating their effect on their welfare (Castro Silva et al., 2023).

### 1.4. Aim

JD–R is a broad and comprehensive model involving a wide range of factors that can affect employees’ work, satisfaction, and health. However, it is also flexible enough to apply to any organisation or profession. It proposes that all dimensions of job demands/resources will not have equal importance for every profession, thus “physical demands will be more relevant for a farmer, and mental demands more relevant for a teacher” (Demerouti & Bakker, 2011). For this reason, we wanted to examine which factors are particularly important in the teaching profession (GE vs. SEN teachers), as well as whether the predictive power of job resources as protective factors and job demands as risk factors for developing burnout can be confirmed. In this way, measures aimed at preventing burnout at work can be focused on the most important risk/protective factors for the teaching profession.

The aim of this study was to examine and compare the potential relationship between the intensity of Emotional exhaustion (EE), Depersonalisation (DP), and Personal accomplishment (PA) and Job demands/Job resources in a population of SEN and GE teachers. Two hypotheses were put forward: H1—job demands (Cognitive/Quantitative, Emotional, and Physical job demands, Changes, Role conflict, and Work environment) are positively correlated with EE and DP, and negatively with PA, i.e., represent potential “risk factors”, and H2—job resources (Job control, Support from managers, and Support from colleagues) are negatively correlated with EE and DP, and positively with PA, i.e., represent potential “protective factors” in the development of workplace burnout.

## 2. Materials and Methods

The study is a part of an ongoing research project exploring workplace burnout in SEN teachers and primary school teachers within general education in Serbia. The research was approved by the Ethics Committee, Faculty of Medical Sciences, University of Kragujevac N 01–1382. Participation in the research was anonymous and voluntary, with each participant being able to withdraw from the study if they felt the questions disturbed their mental well-being and/or upset them in any way. All participants signed informed consent.

### 2.1. Sample

*Inclusion criteria* for the participation in the study were as follows: to be a SEN teacher working in a primary school for students with learning difficulties (in specific, with intellectual or cognitive impairment) or teachers who work in primary schools of general education, and to be from 25 to 60 years of age, with at least one year of work experience in the profession (to avoid the impact of the employees’ initial adaptation to new elements of work).

The sample recruited 116 SEN teachers, working in all eight special educational needs schools for learners with intellectual or cognitive impairment in the Belgrade region (M_age_ = 41.88, SD = 8.07) with average work experience M = 15.28, SD = 7.74. The selection of general schools was randomised within the same region as for the SEN schools, with the sample of 145 GE teachers (M_age_ = 41.47, SD = 9.09) with average work experience M = 13.97, SD = 9.28.

In order to enable a more precise comparison between the said groups, the sample of GE teachers was balanced according to the representation of the main demographic variables in the SEN group (Table 1). There was no difference in gender (*χ*^2^(1) = 0.762, *p* = 0.476), age (*t*(259) = 0.381, *p* = 0.704), education level (*χ*^2^(2) = 2.645, *p* = 0.266), seniority (*t*(259) = 1.219, *p* = 0.224), work contract (*χ*^2^(1) = 1.385, *p* = 0.239), nor marital status (*χ*^2^(2) = 1.124, *p* = 0.570).

### 2.2. Instruments

To investigate the influence of job characteristics, the *Job Characteristics Questionnaire* was employed. The original version of the scale comprises 46 items (Radošević et al., 2018), while in this study we used the shortened version of the scale, which displayed a more stable factor structure and was standardised for Serbian population (Popov et al., 2025). It is a five-point Likert-type scale (ranging from 1—*almost never* to 5—*almost always*), including nine dimensions examining different Job demands—*Cognitive* and *Quantitative demands*, *Emotional demands*, *Physical demands*, *Role conflict*, *Changes*, and *Work environment*, and Job resources—*Job control*, *Support from managers*, and *Support from colleagues*. Quantitative demands are measured via work pace and deadlines, while Cognitive demands are tested through a great amount of information and focus on work. Emotional demands, among other things, relate to relationships and situations in which an employee hides and/or suppresses their emotions. Physical demands refer to a physical strain exposure, standing and walking during a longer period in a workday, and so on. Job control is tested with items denoting a possibility for employees to affect different aspects of work, such as number of breaks, manner of conducting work, and so forth. Role conflict subsumes situations in which an employee is assigned tasks that are beyond the scope of work or tasks they are not sufficiently competent in, along with the level of understanding of what they are expected to do in the workplace. Workplace changes are tested through a possibility for employees to anticipate and monitor changes in their organisation. Work environment measures conditions under which an employee works (e.g., occupational noise exposure, lighting, and air conditioning). Support from managers relates to encouragement, rewarding, and cooperativeness of their manager, whereas Support from colleagues refers to interpersonal relationships and a positive working atmosphere. In this study we administered the instrument with all nine dimensions. Since there is no equal distribution of items per dimension, we calculated mean values for each dimension for comparability purposes. Internal consistency coefficients for items on the scale ranged from 0.56 to 0.87 in early studies (Popov et al., 2025). In our study, the internal consistency values for the nine subfactors ranged from 0.69 to 0.86.

Three dimensions of the burnout construct were displayed through scores on the *Maslach Burnout Inventory*—MBI–HSS (Maslach et al., 2016), i.e., its standardised Serbian version, which measures the frequency and intensity of the said three dimensions in individuals working within humanities and helping professions. The scale consists of 22 items in total, ranging from 0—*never* to 6—*daily*, with three subscales testing the following: Emotional exhaustion—a feeling of exhaustion of emotional and physical resources, with nine items and values on the subscale from 0 to 35; Depersonalisation—a negative, excessively indifferent response to different aspects of work and a sense of detachment from people in the workplace, with five items and values on the subscale from 0 to 30; and Personal accomplishment—a sense of competence and accomplishment in working with people, with eight items and values on the subscale from 0 to 48. The scale authors’ recommendation is that the subscales should not be merged into one burnout scale, i.e., that subscales scores should not be combined to form a single burnout score, but calculated and interpreted separately (Maslach et al., 2016). Accordingly, internal consistency of the subscales in this study ranged from α = 0.71 for the DP subscale, over α = 0.82 for the PA subscale, to α = 0.91 for the EE subscale.

A demographic questionnaire was also used, including questions about gender, age, education, seniority, and types of work contract.

### 2.3. Statistical Analyses

The data were processed by IBM SPSS Statistics 26. A normal distribution of scores on the main variables was examined by means of the skewness/kurtosis criteria of ±2. Differences in scores were computed by independent samples *t*-test and ANOVA, while Pearson’s coefficient was used for correlations. Regression analysis was employed to test three separate predictive models for each dimension of the MBI scale independently.

## 3. Results

Descriptive statistics data pertaining to burnout and work characteristics are presented in Table 2 for both tested groups.

In both groups all mean values were within the skewness and kurtosis limit of ±2, suggesting normality of data distribution (Hair et al., 2010).

No difference in scores was found between the groups on either of the three MBI dimensions of (see Table 2). However, there were differences in a few job resources/demands. GE teachers perceived Cognitive/Quantitative, and Emotional demands, as well as Support from colleagues as more prominent. SEN teachers, on the other hand, reported higher scores on Physical demands.

### 3.1. Burnout Dimensions Correlates

Since there was a difference between the two groups in their perception of JD–R, correlations were calculated for each group separately (see Table 3). Both groups reported mainly lower or moderate correlations, with several strong correlation coefficients.

Regarding the remaining control variables, the following differences were reported^2^. According to gender, only a higher level of EE in female GE teachers was detected (*t*(143) = 2.360, *p* = 0.02). According to the type of work contract, employees with indefinite employment contracts reported higher values on EE in the GE group (*t*(143) = 4.380, *p* < 0.001), and DP (*t*(143) = 2.124, *p* < 0.05), as well as EE (*t*(114) = 2.670, *p* < 0.01), and DP in the SEN group (*t*(114) = 2.018, *p* < 0.05). According to the education level, differences were found only with the bachelor’s degree teachers, who reported higher levels on EE (*t*(140) = 2.956, *p* < 0.01) and DP (*t*(140) = 2.146, *p* < 0.05) in contrast to postgraduate degrees.

Importantly, certain Job demands/resources were very strongly intercorrelated (see Table 4). Specifically, Support from managers, Support from colleagues, and Changes were very strongly intercorrelated in both groups.

### 3.2. Models of Prediction

Finally, hierarchical regression was performed, whereby hypothetical models of burnout dimensions were tested in SEN and GE groups. According to these, Job demands were hypothesised to be risk factors and Job resources to be protective factors.

Although initial predictive models covering all aspects of work were found significant in SEN teachers (EE—*F*(9,106) = 12.495, *p* < 0.001; DP—*F*(9,106) = 5.569, *p* < 0.001; PA—*F*(9,106) = 4.796, *p* < 0.001), only a few variables significantly contributed to prediction variance for all three dimensions included in the study. Upon exclusion of the variables not contributing greatly to prediction variance by means of stepwise regression analysis (see Table 5), the obtained model predicted 48% of the EE variance (*F*(4,111) = 28.019, *p* < 0.001) whose positive predictors were Emotional demands, Work environment and Changes, and negative Support from colleagues (the stronger support, the lower level of exhaustion).

The model including Work environment and Changes (see Table 6), positively predicted 25% of the Depersonalisation variance (*F*(2,113) = 19.793, *p* < 0.001).

Lastly (see Table 7), the model including Changes, Job control, and Cognitive/Quantitative demands, with 24% of prediction variance, was found to be a good predictor of a sense of Personal accomplishment (*F*(3,112) = 13.073, *p* < 0.001).

Predictive models covering all work aspects were found significant in GE teachers too. Upon exclusion of dimensions that were not shown to be significant predictors (see Table 8), 47% (Adjusted R^2^) of the EE variance was significantly predicted (*F*(4,140) = 32.310, *p* < 0.001) by a model featuring Support from colleagues, Seniority (the longer work experience, the higher level of burnout), and Job control.

The model (see Table 9) with Changes and Cognitive/Quantitative demands predicted 28% of the depersonalisation variance (*F*(3,141) = 19.642, *p* < 0.001).

Finally, 17% of the personal accomplishment variance was significantly predicted (*F*(3,141) = 10.615, *p* < 0.001) by the model (see Table 10) including Job control and Cognitive/Quantitative demands.

**Figure 1:**
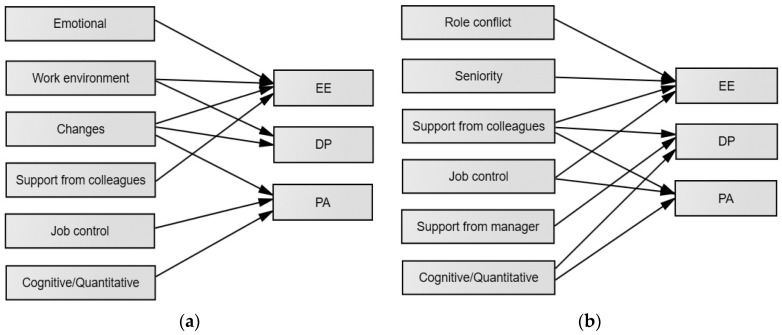
Predictors of burnout dimensions in SEN teachers (**a**) and GE teachers (**b**).

Due to the fact that the three dimensions are not recommended to be merged into a single construct (of burnout), we were not able to create a single all-inclusive structural model. Nonetheless, based on the data and for clarity purposes, we graphically presented all work aspects predicting the three burnout dimensions in SEN and GE teachers in parallel (see Figure 1).

## 4. Discussion

This study detected no differences in the prominence of burnout dimensions between the SEN and GE teachers in the sample. Although there are studies reporting similar results (Saloviita & Pakarinen, 2021), there is a large body of research suggesting that SEN teachers experience a higher level of exhaustion and decline in work efficiency in comparison to GE teachers (De Stasio et al., 2017; Stempien & Loeb, 2002). A possible reason underlying such findings is that there are differences in formal education systems and duration of teacher training across the regions in the world. In Serbia, SEN teachers are trained to work with learners needing intensive support during their development for four or five years of formal education. Moreover, research into burnout syndrome in SEN teachers in Serbia depending on the type of disability (students with sensory disabilities, intellectual disabilities, disorders in social behaviour, motor skill disorders) show that SEN teachers working with learners with intellectual disabilities report the lowest levels of burnout (V. Jovanović et al., 2019).

Additionally, GE teachers may be experiencing burnout to a higher degree nowadays (V. R. Jovanović et al., 2021). GE teachers teach several class groups with a greater number of students than SEN teachers (Saloviita & Pakarinen, 2021). Numerous changes in the education system of general education in Serbia in the last few years, such as inclusion of learners with disabilities, add another source of stress in GE teachers. Obligation to keep creating and documenting new syllabi and EPs (Forlin, 2001), and to adapt subject matter content to learners with disabilities, further expands their workload and places them under time pressure. Accordingly, our study confirms that GE teachers thus perceive higher levels of cognitive and quantitative job demands. This can result in feelings of greater emotional demands in these teachers and concerns about their competence to work with learners needing additional support (Saloviita & Pakarinen, 2021).

Finally, SEN teachers reported to have more physical strains during work in comparison to GE teachers. This finding is expected because the participants working in SEN schools are focused not only on educational work but also on providing care to enable students to carry out self-care activities.

### 4.1. Prediction of Job Burnout via the JD–R Model

Both research hypotheses were confirmed, with job demands being positive predictors and resources negative predictors of burnout dimensions. Since every profession has its specific spectre of work demands and resources related to stress development in the workplace (Demerouti & Bakker, 2011), in this study we aimed to design a factor model predicting workplace burnout dimensions in SEN and GE teachers and to compare them.

#### 4.1.1. Emotional Exhaustion

Emotional demands, Work environment, Changes, and Support from colleagues were singled out as the leading predictive factors of emotional exhaustion in SEN teachers, while Support from colleagues, Role conflict, Job control, and Seniority played their role in GE teachers.

Changes, Role conflict, and Job control (which is compromised when there are role conflict and frequent changes in the workplace) are significant risk factors for the development of emotional exhaustion in the teachers from this study and may be the consequence of recurrent education reforms in Serbia from 2000 onwards. Researchers highlight that frequent education reforms generate pressure and negative emotional responses of teachers (Skaalvik & Skaalvik, 2009; Tsang & Kwong, 2016). Job control is often underscored as key factor in preventing SEN teachers from leaving their job (Stempien & Loeb, 2002). Teachers in general education perceive that their responsibilities have increased (particularly administrative duties) and that job control is restricted to classroom work only, which makes them insufficiently prepared to implement policy changes and thus accept them (Pantić & Čekić-Marković, 2012). The traditional role of GE teachers has changed, and they face numerous roles nowadays (Pillay et al., 2005), such as active participation in inclusive education. These changes and job enlargement are likely to be associated with Role conflict and Role ambiguity in GE teachers. Garwood et al. (2018) found role conflict among coworkers to significantly contribute to emotional exhaustion of teachers, as they believed to have been working hard towards unclear goals. Even when an employee has optimum control over their job, if they are unclear about their duties, autonomy would not create a sense of freedom to make decisions but may rather intensify anxiety and frustration (Soini et al., 2019).

Accordingly, correlations between Job control, Changes, and Role conflict from this study, along with overlaps in content of some items on these subscales, point to very similar impacts on EE in both groups of teachers, who fundamentally feel lack of control and autonomy as a consequence of recurrent changes in duties and hence role conflict. Therefore, the result showing that seniority was shown to be a risk factor in GE teachers comes as no surprise, because stress caused by longer work in changeable conditions (*r* = 0.276, *p* < 0.01) and lack of control (*r* = 0.225, *p* < 0.01) has accumulated over time, which is supported by the correlation between years of work experience and these two aspects of work.

Support from colleagues was found to be a significant protective factor in burnout prevention in both tested groups, which was the case in other studies as well (Brouwers et al., 2001; Drobac & Micic, 2025; Hsieh et al., 2022; Skaalvik & Skaalvik, 2016; Stempien & Loeb, 2002; Zeng et al., 2024). Collegial atmosphere can help teachers to learn from other teachers and to be more engaged and motivated in class, and consequently more satisfied. Sharing stressful events with others and communicating the corresponding emotions are likely to facilitate positive psychological adjustment, thereby making employees more resilient to workplace burnout (Stanley & Sebastine, 2023).

Lastly, Work environment was also singled out in SEN teachers, which was found to be an important aspect of work in other studies as well (Drobac & Micic, 2025; Hsieh et al., 2022; Cumming et al., 2021). SEN teachers who experienced more supportive working conditions (i.e., stronger logistical resources) reported less emotional exhaustion and stress and felt greater self-efficacy for instruction (Cumming et al., 2021). Work environment is regarded as a burnout risk factor in SEN teachers because working conditions, workplace equipment, and availability of work materials are definitely important for every profession, but they are crucial in the teaching profession. In comparison to GE teachers, who are mainly focused on learning outcomes, SEN teachers are also focused on solving problems deriving from learning difficulties, with accompanying specific teaching aims and classroom equipment, classroom layout, etc.

#### 4.1.2. Depersonalisation

Within the SEN group, depersonalisation was found to be predicted by the same organisational factors that were predictors of emotional exhaustion, i.e., Work environment and Changes. The presence of the same predictors suggests a strong connection between emotional exhaustion and depersonalisation. According to Maslach, emotionally exhausted teachers are struggling to face the psychological pressure of the workplace in this stage, thereby developing a passive relation with learners and colleagues (Maslach et al., 2001).

Significant depersonalisation factors in GE teachers were Changes, Support from colleagues, and Cognitive demands. Since Support from colleagues was also found to be important for reducing the risk of emotional exhaustion development in GE teachers, we may assume that this factor is relevant for further development of burnout effects, thus increasing the risk of intensified emotional exhaustion with more profound consequences in the case of lack of this support. Changes, as previously stated, are likely to be connected with Role conflict and Job control, which are singled out in the EE model. Finally, when increased cognitive demands are added to emotional exhaustion, teachers are likely to withdraw and depersonalise in response, which is in line with Maslach’s model.

#### 4.1.3. Personal Accomplishment

Changes, Job control, and Cognitive/Quantitative demands predicted a sense of personal accomplishment in SEN teachers, whereas in GE teachers those were Job control, Support from colleagues, and Cognitive/Quantitative demands. Besides the implications stated with the previous dimensions, we should emphasise that acknowledgment of accomplishment by immediate environment, together with social support, was shown to have the potential for self-actualisation in teachers (V. R. Jovanović et al., 2021). Additionally, feedback on performance and support from colleagues, or else managers, may alleviate the effects of high job demands, especially through engagement in the workplace (Pakdee et al., 2025).

### 4.2. Demographic Variables

Although demographic variables have been commonly underscored as significant demographic burnout predictors, in this study they were not shown to wield direct influence on burnout development. Seniority stood out as the most important variable, whereby longer work experience was a difference factor for the development of emotional exhaustion in GE teachers. This finding is in accordance with the burnout concept regarded as a state of exhaustion caused by long-term engagement in emotionally demanding situations (Rostami et al., 2015), as well as the thesis that burnout often appears early and late in teachers’ career (Ozdemir, 2007). Elder teachers have undergone various changes in the workplace through education reforms in Serbia, which requires new skills and knowledge, so work experience can lead to accumulated long-time demands and stress (V. R. Jovanović et al., 2021). In our study this was supported by a positive correlation with changes in the workplace and lack of control. Sex is a variable that commonly yields mixed results (Rostami et al., 2015), so it apparently depends on various socio-cultural factors, from type of employment contract, workload, to cultural milieu. Regarding the type of employment contract, the results of our study suggest that teachers with indefinite employment contract are prone to burnout to a larger extent, irrespective of the group (SEN, GE teachers). This finding may be interpreted through their association with the participants’ age and years of work experience, as teachers with fixed-term employment contracts tend to be younger persons. GE teachers with a bachelor’s degree reported higher levels of burnout than teachers with a postgraduate degree. The obtained data may be connected with higher salaries, potential work assignments, or their status in the school.

### 4.3. Study Limitations and Directions for Future Research

The main limitations are that it is a cross-sectional study and that we used self-assessment instruments to collect data. Therefore, future research may include some of the *outcomes* of burnout syndrome (absence from work, fluctuation, presence of psychophysical symptoms).

According to the JD–R Model and later research (Bakker et al., 2014), job demands are more important predictors of burnout than lack of job resources, i.e., “job resources may *buffer* the impact of job demands on job strain, including burnout” (Demerouti et al., 2001; Demerouti & Bakker, 2011). The results obtained in this study form the basis for testing a more specific model, in which job resources would be mediating variables between job demands and burnout dimensions. In a similar vein, Support from managers was not shown to be a direct predictor of either burnout dimension. Although initial analyses showed this factor to be strongly correlated with all three dimensions in both groups, in subsequent models, where different aspects of work strongly correlating with Support from managers were introduced (primarily, Support from colleagues, Changes and Job control), the significance of this variable was downplayed. These are exactly the conditions for defining the existence of a *mediating effect* of a variable, which makes it a solid ground for further investigation of a mediating role of job resources. Support from superiors is associated with teachers’ autonomy and engagement in the process of decision making (Maslach & Leiter, 2016). This type of support represents a significant predictor of self-efficacy and diminishes teachers’ desire to change their job (Skaalvik & Skaalvik, 2016). Absence of support from managers can therefore be a significant burnout factor, but also acts as a mediator of other aspects of work such as job demands. Finally, this potential model should definitely feature some of the individual characteristics and/or resources, such as resilience, self-efficacy, and extraversion, which were found to be common mediators between job characteristics and outcomes of teachers work (Bakker et al., 2014; V. R. Jovanović et al., 2021; Richards et al., 2018; Taris et al., 2017).

## 5. Conclusions

The current study points to the need for further and in-depth understanding of organisational factors through a model of demands and resources that may influence burnout coping strategies. Teachers exposed to frequent changes in the workplace, less control over their work, and higher level of role conflict are more susceptible to workplace burnout. Support from colleagues is a protective factor of all burnout phases and an important resource in its prevention, and together with support from managers it may play a significant mediating role between job demands and burnout dimensions. Formulation of complex organisational predictive models would facilitate taking targeted actions towards enhancing protective and reducing risk factors of teachers’ burnout in different fields of education. By adopting the JD–R Model as a theoretical framework, researchers can gain a deeper understanding of the mechanisms underlying the relation between work capacity and its predictors, thereby offering insights into prevention and intervention strategies for enhancing work efficacy among teachers (Hlado & Harvankova, 2024).

The practical contribution of the current research lies in the finding that specific job demands pose a risk for the emergence and development of burnout syndrome in the SEN and GE teacher groups. In addition, research significance lies in the identification of similar challenges posed to teachers, irrespective of the education type (SEN and GE teachers). If we were to summarise our participants’ massage to the policy, it would be, “*I am constantly going through changes. I feel like I am not always in control. I need support*”. Accordingly, the planning of strategies aimed at preserving mental health in teaching profession needs to include burnout prevention measures timely and particularly during the implementation of education policy changes. The prevention measures should be reliant on organisational resources, such as reinforcing the mutual support among employees.

## Figures and Tables

**Table 1:** Sociodemographic characteristics of the sample.

		SEN	GE
Sex	Female	102 (87.9%)	122 (84.1%)
	Male	14 (12.1%)	23 (15.9%)
Education level	College of applied studies	0	3 (2.1%)
	Graduate studies	105 (90.5%)	126 (86.9%)
	Postgraduate studies	11 (9.5%)	16 (11.0%)
Work contract	Indefinite employment	90 (77.6%)	102 (70.3%)
	Fixed-term employment	26 (22.4%)	43 (29.7%)
Marital status	Married/in a relationship	77 (66.4%)	87 (60.0%)
	Single	29 (25.0%)	43 (29.7%)
	Other	10 (8.6%)	15 (10.4%)

**Table 2:** Descriptive data and group differences.

	Group	N	M	SD	Min/ Max	Skewn.	Kurt.	*t*
Cognitive/ Quantitative demands	SEN	116	3.69	0.63	2/5	−0.218	−0.068	*t*(259) = −3.942 **
	GE	145	3.99	0.59	2/5	−0.524	0.651	
Emotional demands	SEN	116	3.88	0.65	2/5	−0.224	−0.560	*t*(259) = −2.382 *
	GE	145	4.06	0.57	2.7/5	−0.335	−0.372	
Physical demands	SEN	116	2.61	0.99	1/5	0.250	−0.593	*t*(259) = 3.262 **
	GE	145	2.26	0.73	1/4	0.068	−0.760	
Job control	SEN	116	3.43	0.63	2/5	−0.104	−0.495	*t*(259) = 1.547
	GE	145	3.32	0.51	2/4.5	−0.344	−0.382	
Support from managers	SEN	116	3.78	0.94	1.2/5	−0.600	−0.330	*t*(259) = −1.934
	GE	145	3.98	0.79	2.5/5	−0.432	−0.609	
Support from colleagues	SEN	116	3.78	0.77	1.5/5	−0.374	−0.136	*t*(259) = −2.727 **
	GE	145	4.02	0.62	2/5	−0.572	0.309	
Role conflict	SEN	116	2.60	0.49	1.6/4.2	0.159	0.191	*t*(259) = 0.325
	GE	145	2.58	0.50	1.4/3.6	0.209	−0.412	
Changes	SEN	116	2.37	0.80	1/4	−0.020	−1.16	*t*(259) = −0.898
	GE	145	2.45	0.63	1/4	−0.121	−0.502	
Work environment	SEN	116	2.64	0.92	1/4.8	0.104	−0.601	*t*(259) = −0.464
	GE	145	2.69	0.77	1/4.8	−0.001	−0.469	
Emotional exhaustion	SEN	116	22.43	13.71	1/54	0.253	−0.967	*t*(259) = −0.015
	GE	145	22.46	11.79	0/48	0.159	−0.786	
Depersonalisation	SEN	116	4.72	4.76	0/21	1.122	0.516	*t*(259) = −1.384
	GE	145	5.54	4.78	0/21	1.065	0.820	
Personal accomplishment	SEN	116	38.53	7.19	18/48	−0.632	−0.304	*t*(259) = 0.285
	GE	145	38.28	6.55	16/48	−0.968	1.06	

Note: * *p* < 0.05, ** *p* < 0.01.

**Table 3:** Correlations of JD–R and control variables with burnout dimensions scores.

**SEN Teachers**	**EE**	**DP**	**PA**
Cognitive/Quantitative demands	0.058	−0.080	0.180
Emotional demands	0.402 **	0.190 *	−0.162
Physical demands	0.306 **	0.213 *	−0.124
Job control	−0.405 **	−0.284 **	0.404 **
Support from managers	−0.606 **	−0.362 **	0.345 **
Support from colleagues	−0.527 **	−0.407 **	0.376 **
Role conflict	0.443 **	0.396 **	−0.208 *
Changes	0.617 **	0.473 **	−0.382 **
Work environment	0.485 **	0.382 **	−0.252 **
Age	0.191 *	0.086	−0.041
Seniority	0.193 *	0.072	−0.044
**GE Teachers**	**EE**	**DP**	**PA**
Cognitive/Quantitative demands	0.212 *	0.220 **	0.147
Emotional demands	0.257 **	0.068	0.007
Physical demands	0.279 **	0.202 *	−0.036
Job control	−0.377 **	−0.253 **	0.337 **
Support from managers	−0.546 **	−0.527 **	0.319 **
Support from colleagues	−0.503 **	−0.407 **	0.307 **
Role conflict	0.493 **	0.411 **	−0.144
Changes	0.481 **	0.471 **	−0.284 **
Work environment	0.449 **	0.295 **	−0.265 **
Age	0.368 **	0.173 *	−0.084
Seniority	0.462 **	0.280 **	−0.102

Note: * *p* < 0.05, ** *p* < 0.01.

**Table 4:** Job resources/demands intercorrelations.

**SEN Teachers**	**EMO**	**PHY**	**CON**	**SM**	**SC**	**RC**	**CH**	**WE**
Cognitive/Quantitative	0.215 *	0.364 **	0.036	−0.048	−0.030	0.000	0.075	0.161
Emotional	1	0.282 **	−0.176	−0.292 **	−0.147	0.307 **	0.289 **	0.262 **
Physical	0.282 **	1	−0.332 **	−0.260 **	−0.188 *	0.388 **	0.272 **	0.510 **
Job control	−0.176	−0.332 **	1	0.484 **	0.485 **	−0.303 **	−0.388 **	−0.372 **
Support from managers	−0.292 **	−0.260 **	0.484 **	1	0.723 **	−0.537 **	−0.820 **	−0.415 **
Support from colleagues	−0.147	−0.188 *	0.485 **	0.723 **	1	−0.432 **	−0.680 **	−0.293 **
Role conflict	0.307 **	0.388 **	−0.303 **	−0.537 **	−0.432 **	1	0.582 **	0.530 **
Changes	0.289 **	0.272 **	−0.388 **	−0.820 **	−0.680 **	0.582 **	1	0.451 **
Work environment	0.262 **	0.510 **	−0.372 **	−0.415 **	−0.293 **	0.530 **	0.451 **	1
**GE Teachers**	**EMO**	**PHY**	**CON**	**SM**	**SC**	**RC**	**CH**	**WE**
Cognitive/Quantitative	0.320 **	0.272 **	−0.010	−0.126	−0.015	0.262 **	0.197 *	0.220 **
Emotional	1	0.178 *	−0.183 *	−0.118	0.024	0.191 *	0.281 **	0.392 **
Physical	0.178 *	1	−0.088	−0.178 *	−0.159	0.334 **	0.313 **	0.373 **
Job control	−0.183 *	−0.088	1	0.360 **	0.295 **	−0.290 **	−0.350 **	−0.369 **
Support from managers	−0.118	−0.178 *	0.360 **	1	0.628 **	−0.566 **	−0.704 **	−0.502 **
Support from colleagues	0.024	−0.159	0.295 **	0.628 **	1	−0.368 **	−0.434 **	−0.310 **
Role conflict	0.191 *	0.334 **	−0.290 **	−0.566 **	−0.368 **	1	0.564 **	0.497 **
Changes	0.281 **	0.313 **	−0.350 **	−0.704 **	−0.434 **	0.564 **	1	0.541 **
Work environment	0.392 **	0.373 **	−0.369 **	−0.502 **	−0.310 **	0.497 **	0.541 **	1

Note: * *p* < 0.05, ** *p* < 0.01.

**Table 5:** Model of emotional exhaustion in the SEN group.

Model	Unstandardised Coefficients		Standardised Coefficients	*t*	Sig.
	B	Std. Error	Beta		
(Constant)	−1.480	10.089		−0.147	0.884
Emotional	4.698	1.487	0.224	3.159	0.002
Work environment	3.370	1.129	0.227	2.984	0.003
Changes	5.079	1.709	0.296	2.973	0.004
Support from colleagues	−4.026	1.627	−0.226	−2.475	0.015

**Table 6:** Model of Depersonalisation in the SEN group.

Model	Unstandardised Coefficients		Standardised Coefficients	*t*	Sig.
	B	Std. Error	Beta		
(Constant)	−3.491	1.373		−2.543	0.012
Changes	2.249	0.540	0.378	4.165	0.000
Work environment	1.093	0.469	0.211	2.332	0.021

**Table 7:** Model of Personal accomplishment in the SEN group.

Model	Unstandardised Coefficients		Standardised Coefficients	*t*	Sig.
	B	Std. Error	Beta		
(Constant)	25.374	5.465		4.643	0.000
Changes	−2.567	0.797	−0.286	−3.222	0.002
Job control	3.248	1.004	0.286	3.234	0.002
Cognitive/ Quantitative demands	2.196	0.938	0.191	2.340	0.021

**Table 8:** Model of emotional exhaustion in the GE group.

Model	Unstandardised Coefficients		Standardised Coefficients	*t*	Sig.
	B	Std. Error	Beta		
(Constant)	29.954	9.289		3.225	0.002
Support from colleagues	−5.098	1.298	−0.268	−3.927	0.000
Role conflict	7.117	1.579	0.302	4.507	0.000
Seniority	0.393	0.081	0.310	4.832	0.000
Job control	−3.290	1.537	−0.141	−2.140	0.034

**Table 9:** Model of depersonalisation in the GE group.

Model	Unstandardised Coefficients		Standardised Coefficients	*t*	Sig.
	B	Std. Error	Beta		
(Constant)	2.731	3.806		0.718	0.474
Changes	2.469	0.605	0.328	4.078	0.000
Support from colleagues	−2.023	0.607	−0.263	−3.334	0.001
Cognitive/ Quantitative demands	1.224	0.584	0.152	2.096	0.038

**Table 10:** Model of personal accomplishment in the GE group.

Model	Unstandardised Coefficients		Standardised Coefficients	*t*	Sig.
	B	Std. Error	Beta		
(Constant)	10.118	5.318		1.903	0.059
Job control	3.509	1.033	0.270	3.397	0.001
Support from colleagues	2.426	0.841	0.230	2.884	0.005
Cognitive/Quantitative	1.701	0.842	0.154	2.019	0.045

## Data Availability

The data that support the findings are unavailable due to privacy restrictions.

## Notes

1. Although depersonalisation is often replaced by the term *cynicism* (emphasising attitude toward work in general, not only toward people) in this paper we will use the original term, because among teachers, burnout is most often reflected in relationships with students, parents, and colleagues.

2. Due to lack of space, only significant differences are reported in the text. Contact the corresponding author for complete data concerning control variables.
